# Effects of ACL Reconstructive Surgery on Temporal Variations of Cytokine Levels in Synovial Fluid

**DOI:** 10.1155/2016/8243601

**Published:** 2016-05-29

**Authors:** Marco Bigoni, Marco Turati, Marta Gandolla, Paola Sacerdote, Massimiliano Piatti, Alberto Castelnuovo, Silvia Franchi, Massimo Gorla, Daniele Munegato, Diego Gaddi, Alessandra Pedrocchi, Robert J. Omeljaniuk, Vittorio Locatelli, Antonio Torsello

**Affiliations:** ^1^Orthopedic Department, San Gerardo Hospital, University of Milano-Bicocca, 20900 Monza, Italy; ^2^Department of Medicine and Surgery, University of Milano-Bicocca, 20900 Monza, Italy; ^3^NearLab, Department of Electronics, Information and Bioengineering, Politecnico di Milano, 20133 Milan, Italy; ^4^Department of Pharmacological and Biomolecular Sciences, University of Milan, 20129 Milan, Italy; ^5^Department of Biology, Lakehead University, Thunder Bay, ON, Canada P7B 5E1

## Abstract

Anterior cruciate ligament (ACL) reconstruction restores knee stability but does not reduce the incidence of posttraumatic osteoarthritis induced by inflammatory cytokines. The aim of this research was to longitudinally measure IL-1*β*, IL-6, IL-8, IL-10, and TNF-*α* levels in patients subjected to ACL reconstruction using bone-patellar tendon-bone graft. Synovial fluid was collected within 24–72 hours of ACL rupture (*acute*), 1 month after injury immediately prior to surgery (*presurgery*), and 1 month thereafter (*postsurgery*). For comparison, a “control” group consisted of individuals presenting chronic ACL tears. Our results indicate that levels of IL-6, IL-8, and IL-10 vary significantly over time in reconstruction patients. In the* acute phase,* the levels of these cytokines in reconstruction patients were significantly greater than those in controls. In the* presurgery* phase, cytokine levels in reconstruction patients were reduced and comparable with those in controls. Finally, cytokine levels increased again with respect to control group in the* postsurgery* phase. The levels of IL-1*β* and TNF-*α* showed no temporal variation. Our data show that the history of an ACL injury, including trauma and reconstruction, has a significant impact on levels of IL-6, IL-8, and IL-10 in synovial fluid but does not affect levels of TNF-*α* and IL-1*β*.

## 1. Introduction

Anterior cruciate ligament (ACL) injuries represent approximately 25% of all knee injuries with an annual incidence of at least 0.8 per 1000 persons aged between 10 and 64 years [1]. Nowadays more than 175,000 ACL reconstructions are performed in the US annually [1, 2]. ACL reconstruction can provide knee stability, permit continued participation in sports, and reduce the incidence of other intra-articular knee injuries, such as meniscal and chondral lesions [3–6]. An association between delaying ACL surgery and the development of subsequent meniscal tears and chondral lesions has been well documented in adults and skeletally immature patients [5, 6]. This consequence could depend on an excessive anterior tibial translation and a rotational instability in the injured knee. Presently, graft choices for primary ACL reconstruction include patellar tendon-bone (BPTB) and hamstring (HT) autografts [7]. Even though BPTB autografts may be better in restoring stability than HT autografts, knee-joint stability restored by ACL reconstruction does not decrease the incidence of posttraumatic osteoarthritis (OA) [4, 7, 8]. Fifty to 60% of patients with ACL-reconstructed knees have radiographic evidence of OA after five years [8, 9]. This observation suggests the importance of other factors in the pathophysiology of posttraumatic OA after ACL injuries, including intra-articular inflammatory reactions. High levels of inflammatory cytokines (interleukins, ILs) such as interleukin-1*β* (IL-1*β*), interleukin-6 (IL-6), and tumor necrosis factor-*α* (TNF-*α*) have been detected in acute and chronic anterior cruciate ligament injured knees, suggesting that cytokines can promote cartilage catabolism through the synthesis of free radicals and metalloproteases (MMPs) and consequently participate in OA development [9–11]. Biochemical changes in the synovial fluid of ACL-damagedknees have been extensively reported, but the evolving joint biochemical processes are not clearly documented [12, 13]. These studies examined cytokines or MMPs in the knee joint in a specific clinical situation such as acute or chronic injury but did not follow these levels longitudinally in individual patients undergoing ACL reconstruction. As there are large variations in absolute levels of synovial fluid cytokines among similarly compromised patients [9, 10], it is critical to track individual patients longitudinally to determine if there are different patient-specific cytokine responses.

In this study, we examined knee joint inflammatory patterns in each patient (i) within 24–72 hours of ACL rupture (*acute*), (ii) 1 month after injury immediately prior to arthroscopic ACL reconstruction surgery (*presurgery*), and (iii) 1 month thereafter (*postsurgery*). In particular, we measured the concentrations of selected pro- and anti-inflammatory cytokines (IL-1*β*, IL-6, IL-8, IL-10, and TNF-*α*) in the synovial fluid. For comparison, a corresponding “control” group consisted of individuals (*n* = 17) presenting chronic ACL tears. The aim of the present study was to better understand how cytokines profiles evolve in the same patient after ACL rupture and after ACL reconstruction with BPTB.

## 2. Methods

### 2.1. Participants

Eight male participants aged between 18 and 40 years (mean: 24.5 ± 7.5 years) presenting ACL injury within 72 hours were recruited by an orthopedic surgeon for the periacute ACL tear group. Inclusion criteria consisted of an isolated ACL tear, or an ACL tear associated with a meniscal tear (not itself requiring surgical treatment) confirmed by a senior orthopedic surgeon using present history, physical examination (including positive Lachman and Pivot Shift tests), magnetic resonance imaging, and a confirmation by arthroscopic examination. In comparison, 17 male patients (mean age: 32.6 ± 9.05 years; 5 patients with isolated ACL tear and 12 patients with associated meniscal tear) were recruited from the local community and selected for a chronic ACL tear (3 months or more from the trauma as previously defined) confirmed clinically, with a MRI and arthroscopic surgery [11]. These chronic ACL tear subjects were pooled as concentrations of IL-1*β*, IL-6, IL-8, IL-10, and TNF-*α* are independent of associated meniscal injury [11]. Individuals were excluded from either group if they had a previous history of knee injury, bone fractures simultaneously at investigated knee sprain, chondral lesion or chondropathy with a grade ≥ II according to Outerbridge classification, inflammatory arthritis, osteochondral lesion, and previous intra-articular injection of steroid or hyaluronic acid or NSAID treatment.

Synovial fluid was collected from participants of the periacute ACL tear group 3 times: (i)* acute*: 24–72 hours from the ACL rupture, (ii)* presurgery*: 1 month after the acute event (when an arthroscopic ACL reconstruction surgery was planned); and (iii)* postsurgery*: 1 month after the surgery. Arthroscopic ACL reconstruction was performed using bone-patellar tendon-bone (BTB) graft by the senior surgeon (Marco Bigoni). Femoral fixation was performed with two bioabsorbable cross-pins using RigidFix (DePuy Mitek, Raynham, MA, USA) and tibial fixation with a bioabsorbable interference screw (9 × 23 mm; DePuy Mitek, Raynham, MA, USA). After surgery, all patients followed the same accelerated rehabilitation protocol focusing on quadriceps recruitment and early gain of full range of motion. In the chronic ACL group, synovial fluid was drawn at the beginning of arthroscopic surgery.

Protocols were approved by the local Human Research Ethical Committee and conformed to the principles outlined in the WMA Declaration of Helsinki. All participants provided written informed consent.

### 2.2. Samples

Synovial fluid was aseptically collected without lavage at the beginning of arthroscopic surgery or during initial clinical reception in the emergency room. Synovial fluid samples, collected in tubes containing EDTA, were immediately centrifuged at room temperature (3,000 g × 10 min) in order to remove cellular debris and the supernatant was stored at −80°C until being assayed [10, 11]. The levels of interleukins (IL-1*β*, IL-6, IL-8, and IL-10) and tumor necrosis factor (TNF-*α*) were measured using specific sandwich enzyme-linked immunosorbent assay (ELISA) according to the manufacturer's instructions (IL-1*β*, IL-10, and TNF-*α*, R&D Systems, Minneapolis, MN; IL-6 and IL-8, eBioscience, San Diego, CA). Detection limits were 2.2 pg/mL, 0.8 pg/mL, 0.5 pg/mL, 2 pg/mL, and 1 pg/mL, respectively, for IL-6, IL-8, TNF-*α*, IL-10, and IL-1*β*.

### 2.3. Statistical Analysis

Statistical analysis was performed using MATLAB (version R2010b; MathWorks). Normality of data distribution was assessed by the Jarque-Bera test. Since data were not normally distributed, nonparametric tests were performed.

When comparing temporal patterns, the Kruskal-Wallis test for statistical difference between 3 groups (i.e.,* acute*,* presurgery*, and* postsurgery*) was performed, which is a nonparametric version of classical one-way ANOVA. In the case of significance, Mann-Whitney test Bonferroni corrected has been performed as* post hoc* analysis. When comparing the periacute ACL tear group of patients and the chronic ACL tear group in the different temporal sampling, the Mann-Whitney test was used, which is the nonparametric formulation of the *t*-test, Bonferroni corrected for multiple comparison. For all statistical tests, *p* values < 0.05 appropriately corrected for multiple comparison where needed were considered to be statistically significant.

For all statistical tests, since they were Bonferroni corrected for multiple comparisons, values of *p* < 0.0167 were considered to be statistically significant.

## 3. Results

### 3.1. Effects of Trauma on Cytokine Levels in Synovial Fluids

Measurement of cytokines levels in samples obtained shortly after ACL tear confirms the picture of an inflammatory acute reaction. In fact, levels of IL-6 were significantly higher (*p* < 0.001) compared to the chronic ACL tear group (Table 1). Similarly, also IL-8 levels were significantly higher (*p* < 0.001) in the acute samples compared to the chronic ACL tear group (Table 1). Compared to chronic ACL group, IL-8 and IL-6 levels were elevated 15- and 11-fold, respectively (Table 1). IL-10 levels were also increased but to a lesser extent (*p* < 0.001), whereas IL-1*β* and TNF-*α* levels were comparable in acute samples and chronic ACL group (Table 1).

### 3.2. Temporal Patterns

**Figure 1:**
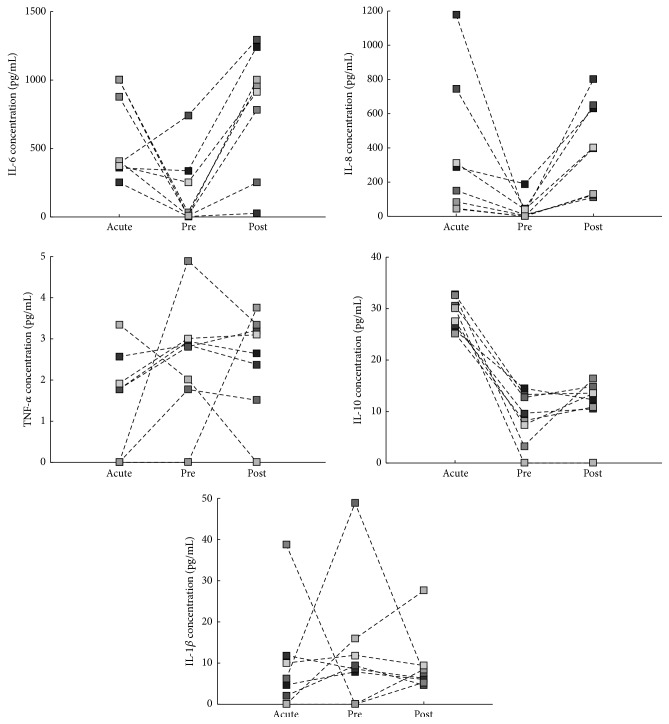
Cytokine levels in the synovial fluid of individual patients after ACL rupture. Squares represent values measured in single subjects (*n* = 8); each subject is represented with different grey values. Acute (less than 72 hours after ACL injury); pre (1 month after the trauma, in or before the arthroscopic ACL reconstruction surgery); post (1 month after the surgery).

**Figure 2:**
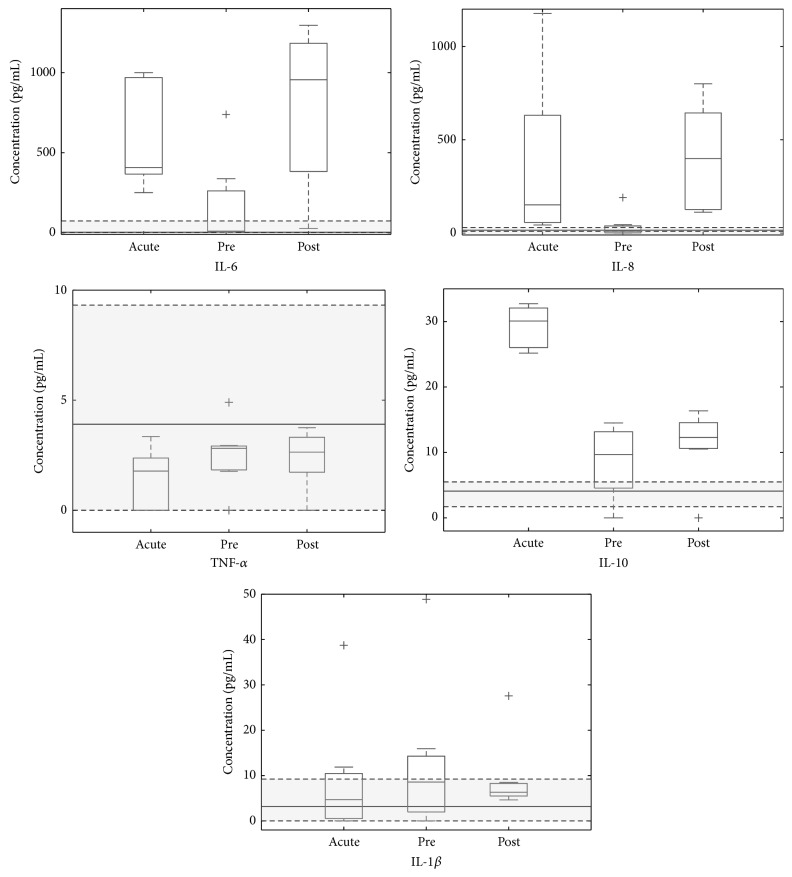
Time-related modifications of cytokine levels compared to chronic ACL group. Cytokine concentrations measured at different time points in the longitudinal group (*n* = 8) are represented in the box-plot. In each box-plot, the box is built within the third (upper bound) and first (lower bound) quartiles (i.e., *Q*_3_,  *Q*_1_); the middle line represents the median. Whiskers represent data maximum (upper whisker) and minimum (lower whisker). + indicates data outliers, defined as data points below *Q*_1_ − 1.5 × (*Q*_3_ − *Q*_1_) or above *Q*_3_ + 1.5 × (*Q*_3_ − *Q*_1_). Grey shaded area represents the interquartile range of the chronic ACL tear group (*n* = 17).

Surgical trauma for ACL reconstruction clearly stimulated a recurrence of the inflammatory picture, causing IL-6, IL-8, and IL-10 concentrations to follow a “V-shaped” trend (i.e., high-low-high values) (Figure 2). Indeed,* acute* and* postsurgery* IL-6, IL-8, and IL-10 levels result in being significantly higher with respect to the chronic ACL tear group (Figure 2). In the presurgery phase, synovial fluid levels of IL-6, IL-8, and IL-10 displayed a rapid decrease (Figure 1), so that 30 days after injury they were in the range of those measured in chronic ACL group (Table 1). IL-1*β* and TNF-*α* levels in each sample of longitudinal group were similar with respect to chronic ACL tear group at any time (Figure 2) and did not follow a specific trend correlated with time after injury (Figure 1). In particular,* post hoc* analysis demonstrated that in the* presurgery* assessment IL-6 and IL-8 concentrations are significantly lower with respect to* acute *assessment (*p* = 0.0111), and in* postsurgery *assessment their levels significantly increased (*p* = 0.0111; *p* = 0.0041). Similarly, we measured a significant decrease of IL-10 levels from the* acute* to* presurgery* samples (*p* < 0.001).

## 4. Discussion

Acute ACL injuries establish an inflammatory reaction that persists chronically after resolution of the acute effusion [10, 14, 15]. Knee-joint inflammatory cytokines can promote cartilage catabolism through the synthesis of free radicals and metalloproteases and develop OA [16, 17]. Moreover, cytokine-mediated inflammatory responses could play an important role in bone tunnel enlargement following ACL reconstruction.

Cytokines as IL-6, IL-8, and IL-1*β* promote osteoclastic activity and can contribute to bone resorption [18]. In a rabbit model, it was described that interarticular bone tunnel healing was slower and less complete in the articular part of the tunnel, suggesting an important role of the synovial environment in the graft integration [19].

Different studies reported high pathological levels of cytokines in the synovial fluid after ACL injury, chronically, and after ACL reconstruction, but to our knowledge the biological natural history of an ACL tear with multiple synovial fluid collections remains uninvestigated [9, 10, 13].

IL-6 and IL-8 are proinflammatory cytokines with an important role in cartilage and bone damage. IL-6 in the joint environment reduces the production of type II collagen, increases the production of MMPS, and is considered to be the key cytokine in the subchondral bone degradation [17, 20]. Moreover,* in vitro* models showed that IL-6 role in the cartilage destruction is markedly potentiated by mechanical injury [21]. IL-6 has the capability to increase the production of inflammatory chemokines, such as IL-8 in synoviocytes and monocytes [22].

IL-8 is a potent chemokine with a key function in the promotion of neutrophil-mediated inflammation and cartilage destruction [23]. These two cytokines present a specific and similar trend in response to the articular events. Immediately after the ACL rupture, IL-6 and IL-8 levels largely increased compared to the chronic ACL tear group. Interestingly, 1 month after IL-6 and IL-8 levels decreased while remaining higher than in the chronic control group. The ACL reconstruction, the presence of foreign bodies and allograft, and the accelerated rehabilitation protocol caused postsurgery levels of IL-6 to increase more than fivefold compared to the presurgery levels. Similarly, also postsurgery levels of IL-8 increased more than tenfold compared to the presurgery levels. This could suggest a role for IL-8 and IL-6 not only in the very early phase of joint inflammation but in all the biological history of a knee with an ACL injury and reconstruction.

IL-10 is a modulator cytokine that contributes to the suppression of the inflammation of the synovial membrane and is endowed with chondroprotective properties. It stimulates the synthesis of type II collagen and aggrecan and antagonizes the release of MMP in chondrocyte [17]. We found high levels of IL-10 in* acute *samples compared to chronic ACL tear group as previously reported [11]. One month later, IL-10 levels decreased more than threefold from the acute ACL injury. The intra-articular stress that follows ACL reconstruction does not stimulate an increase in IL-10 capable of maintaining its levels in time higher than those measured in chronic ACL tear patients. Helmark and colleagues showed an increase in intra-articular IL-10 concentration secondary to specific exercise in patient with knee OA, suggesting an important dependence of the IL-10 production on muscular exercises [24]. Following 1 month of accelerated rehabilitation protocol, we also reported a slight trend towards higher concentrations of IL-10 postoperatively.

Cameron and colleagues observed that their patients could be divided into two subgroups when considering the synovial concentrations of IL-1*β* and TNF-*α* in ACL-deficient knees. One group presented levels of IL-1*β* and TNF-*α* that were high acutely after trauma and decreased to zero in time, whereas the other group had low levels of these cytokines both acutely after trauma and four weeks postoperatively [25]. Based on these data, they speculated that on the basis of their IL-1*β* and TNF-*α* profiles patients could be at different risk of developing posttraumatic OA. However, data demonstrating that patients with high levels of IL-1*β* and TNF-*α* following acute ACL rupture have a higher incidence of OA compared to those presenting low levels of these inflammatory cytokines are still lacking. The data that we have obtained in the present study cannot support Cameron's hypothesis given the small size of the patient population. Even if IL-1*β* and TNF-*α* are two of the most studied and important cytokines in the pathophysiology of OA, more studies to understand their specific role in ACL tear patients are required. Our data showed* acute *levels of IL-1*β* and TNF-*α* comparable to* presurgery* levels and* postsurgery* levels. Essentially, no differences can be reported between longitudinal ACL group and control group, in agreement with previous literature. Indeed, Zysk and coworkers investigated the synovial fluid concentrations of three proinflammatory cytokines (TNF-*α*, IL-1*β*, and IL-6) before ACL surgery (24 ± 7 days after ACL rupture) and 7 days after the surgical operation. They reported that IL-6 levels increased significantly from the pre- to the postoperative measurements whereas IL-1*β* and TNF-*α* concentrations remained unchanged throughout the course of ACL surgery [18].

Interestingly,* in vitro* studies reported that cytokines as IL-1*β* and TNF-*α* have a role in stimulating the synthesis of other cytokines such as IL-6 and IL-8. However different cytokine trends in response to articular events underline the complexity of multiple cascades that could stimulate the synthesis and release of specific cytokines [23].

We are aware of some limitations of the results obtained in this study. We present the data obtained in a limited number of patients, but the prospective design of this pilot study, the strict criteria of samples collection, and storage and analysis and surgical management are very positive aspects. Moreover, we had no access to synovial samples from healthy knees for ethical reasons, and to obviate to this problem we choose to compare data with those of a group of patients with chronic ACL tears.

## 5. Conclusion

This pilot study is to our knowledge the first one describing the temporal evolution of synovial IL-6, IL-8, IL-1*β*, IL-10, and TNF-*α* concentrations from the time of ACL rupture to the postsurgical follow-up in the same patients. These data suggest that ACL injury and ACL surgery have a great impact on IL-6, IL-8, and IL-10 levels in the synovial fluid. TNF-*α* and IL-1*β* levels in synovial fluid followed a different temporal pattern, since they did not increase following acute ACL injury and remained unaltered in time despite the ACL reconstruction surgery.

## Figures and Tables

**Table 1:** Cytokine levels in synovial fluids from longitudinal ACL group and from chronic ACL tear group.

	IL-6 (pg/mL)	IL-8 (pg/mL)	TNF-*α* (pg/mL)	IL-10 (pg/mL)	IL-1*β* (pg/mL)
*Longitudinal ACL group*					
Acute samples	612.19 ± 124.98^*∗*^	362.3 ± 164.60^*∗*^	1.35 ± 0.52	29.09 ± 1.19^*∗*^	9.07 ± 5.18
Presurgery samples	163.34 ± 106.35	38.22 ± 26.05	2.47 ± 0.56	8.82 ± 2.05	12.94 ± 6.35
Postsurgery samples	855.37 ± 183.12^*∗*^	407.48 ± 109.71^*∗*^	2.4 ± 0.48	11.21 ± 2.03^*∗*^	9.42 ± 3.07
*Chronic ACL tear group*					
Samples	58.52 ± 5.86	23.66 ± 1.55	6.15 ± 0.45	4.03 ± 0.21	6.15 ± 0.81

Cytokine concentrations (pg/mL) in the synovial fluid of patients of the longitudinal group (*n* = 8) and chronic ACL tears (*n* = 17). Samples of longitudinal group were further assigned to groups according to the moment of the synovial fluid collection: *acute* samples (less than 72 hours after ACL injury); *presurgery* samples (1 month after the trauma, just before the arthroscopic ACL reconstruction surgery); *postsurgery* samples (1 month after the surgery). Values are expressed as mean ± standard error of the mean (SEM). ^*∗*^Statistically significant periacute samples in comparison with chronic ACL tear group.
